# Smelters and Mortality: Pope et al. Respond

**DOI:** 10.1289/ehp.10447R

**Published:** 2007-09

**Authors:** C. Arden Pope, Douglas L. Rodermund, Matthew M. Gee

**Affiliations:** Department of Economics, Brigham Young University, Provo, Utah, E-mail: cap3@byu.edu

Hidy makes several useful comments regarding our analysis of the mortality effects of a copper smelter strike in the U.S. Southwest (Pope et al. 2007). Regarding issues of atmospheric chemistry, the ambiguities of SO_4_ sampling, and the role of smelter-related trace metals and carbon, Hidy is a well-respected expert, and we do not quibble with these comments. In fact, we briefly addressed the issue of accompanying metals in our discussion, and we are in general agreement that metals, in addition to sulfur oxides and other smelter-related air pollutants, might have played a role in the observed mortality reductions.

With regard to the epidemiologic evidence, one must be careful not to over interpret the small differences in state-specific estimates of strike-period reductions in mortality. A primary statistical inference illustrated in Figure 6 of our article (Pope et al. 2007) is that similar and consistent (not significantly different) mortality decreases were observed across all four Southwest states.

Available data also suggest regional strike-related reductions in SO_4_ concentrations. Based on summary data (Trijonis and Yuan 1978, Table 16), the average (and percent) decrease in SO_4_ concentrations for the urban monitoring sites were 2.7 μg/m^3^ (38%) for Salt Lake City, Utah; 2.3 μg/m^3^ (51%) for Las Vegas, Nevada; 3.6 μg/m^3^ (62%) for Phoenix, Arizona; 2.6 μg/m^3^ (62%) for Maricopa county (near Phoenix); 3.4 μg/m^3^ (67%) for Tucson, Arizona; and 0.1 μg/m^3^ (2%) for Albuquerque, New Mexico. Even the remote sites of White Pine Nevada, Grand Canyon National Park, Arizona, and Mesa Verde National Park, Colorado (near the four corners of Utah, Arizona, New Mexico, and Colorado), observed 1.5 μg/m^3^ (76%), 1.5 μg/m^3^ (60%), and 1.1 μg/m^3^ (57%) reductions in SO_4_ concentrations, respectively. The only notable exception to the region-wide strike-related reductions in SO_4_ concentrations is the negligible reduction in SO_4_ concentrations in Albuquerque [as noted by Hidy and discussed in our article (Pope et al. 2007)]. Regarding Nevada, data from the Las Vegas and White Pine monitoring sites indicated strike-related reductions in SO_4_ similar to those observed at other comparable sites in the region.

Although our analysis of the mortality effects of a copper smelter strike has clear limitations, its unique contribution relates to the relatively simple motivation and natural experimental design. A well-defined 8.5-month copper smelter strike in the 1960s resulted in abrupt, well-documented regional reductions in SO_4_ concentrations and improvements in visibility (Trijonis 1979). Available mortality data indicate a significant strike-period decrease in mortality, even while statistically controlling for time trends, mortality counts in bordering states, and nationwide mortality counts for influenza/pneumonia, cardiovascular, and respiratory deaths (Pope et al 2007). The estimated reduction in mortality is consistent with what would be expected given the average reduction in ambient concentrations of SO_4_ particles and estimated mortality effects from the relevant literature. For example, both the Harvard Six Cities Study (Dockery et al. 1993) and the American Cancer Society cohort studies of long-term air pollution exposure (Pope et al. 2002) reported similar mortality risks associated with fine and SO_4_ particulate pollution. Also, comparable reductions in mortality were observed following the imposition of restrictions on the sulfur content of fuel in Hong Kong (Hedley et al. 2002) and the banning of coal burning in Dublin, Ireland (Clancy et al. 2002).

Finally, it is unclear how the issues addressed by Hidy are likely to confound the estimates of strike-period mortality reduction from a well-defined statistical or epidemiologic perspective. However, these issues do “confound” how we interpret the estimated strike-period reductions in a broader context and to what extent we attribute the observed mortality reductions to smelter-source SO_4_ and related pollutants. We appreciate Hidy’s contributions to our efforts to interpret these and related results.
